# Enhancing Robotic Collaborative Tasks Through Contextual Human Motion Prediction and Intention Inference

**DOI:** 10.1007/s12369-024-01140-2

**Published:** 2024-07-13

**Authors:** Javier Laplaza, Francesc Moreno, Alberto Sanfeliu

**Affiliations:** Institut de Robòtica i Informàtica Industrial de Barcelona, Universitat Politècnica de Catalunya, C/ Llorens i Artigas 4-6, 08024 Barcelona, Catalonia Spain

**Keywords:** Human–robot collaborative task, Human motion prediction, Human intention prediction, Deep learning attention architecture

## Abstract

Predicting human motion based on a sequence of past observations is crucial for various applications in robotics and computer vision. Currently, this problem is typically addressed by training deep learning models using some of the most well-known 3D human motion datasets widely used in the community. However, these datasets generally do not consider how humans behave and move when a robot is nearby, leading to a data distribution different from the real distribution of motion that robots will encounter when collaborating with humans. Additionally, incorporating contextual information related to the interactive task between the human and the robot, as well as information on the human willingness to collaborate with the robot, can improve not only the accuracy of the predicted sequence but also serve as a useful tool for robots to navigate through collaborative tasks successfully. In this research, we propose a deep learning architecture that predicts both 3D human body motion and human intention for collaborative tasks. The model employs a multi-head attention mechanism, taking into account human motion and task context as inputs. The resulting outputs include the predicted motion of the human body and the inferred human intention. We have validated this architecture in two different tasks: collaborative object handover and collaborative grape harvesting. While the architecture remains the same for both tasks, the inputs differ. In the handover task, the architecture considers human motion, robot end effector, and obstacle positions as inputs. Additionally, the model can be conditioned on the desired intention to tailor the output motion accordingly. To assess the performance of the collaborative handover task, we conducted a user study to evaluate human perception of the robot’s sociability, naturalness, security, and comfort. This evaluation was conducted by comparing the robot’s behavior when it utilized the prediction in its planner versus when it did not. Furthermore, we also applied the model to a collaborative grape harvesting task. By integrating human motion prediction and human intention inference, our architecture shows promising results in enhancing the capabilities of robots in collaborative scenarios. The model’s flexibility allows it to handle various tasks with different inputs, making it adaptable to real-world applications.

## Introduction

**Fig. 1:**
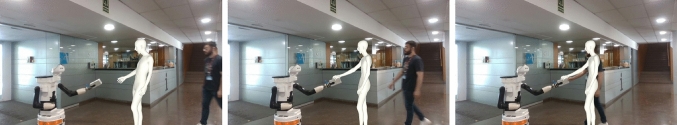
Visual representation of the prediction being used by the robot. We utilized the SMPL parametric model [1] to provide a realistic visualization of the 3D human body of the user during a human–robot handover. In the final frame, the user reached a close position with respect to the predicted pose

Our research argues that by integrating task context, human intention, and motion prediction, robots can significantly enhance the quality of interactions in collaborative operations with humans. 3 In the domain of robotics, precise human motion prediction is of paramount importance for collaborative tasks where humans and robots operate in close proximity. We define motion prediction as the task of predicting a feasible future human motion given one or more previous frames of past human poses. In Fig. 1 we can see how a robot can be using the prediction of the human body while trying to approach him). In such scenarios, accurate prediction is crucial for ensuring safe and efficient interactions. Conversely, longer predictions could prove valuable for tasks like action recognition. However, a significant challenge in developing effective human motion predictors that can account for the presence of a robot nearby is the lack of datasets that encompass human–robot interactions and incorporate external factors or task-specific information. This limitation impedes the comprehensive advancement of human motion prediction models in real-world collaborative environments. To address this issue, there is an urgent need for the creation of new datasets that capture the complexities of human–robot interaction and can serve as training and evaluation resources for deep learning models focused on human motion prediction in such scenarios.

In this study, we address the challenge of human motion prediction in human–robot interaction (HRI), where accuracy in predictions is of upmost importance to mitigate collision risks during close interactions. Consequently, we choose to employ the standard $L_2$ metric for both training and evaluating our proposed neural network model. However, our approach extends beyond the sole use of the $L_2$ metric. We incorporate supplementary loss functions to effectively harness contextual insights. The model does not only account for the contextual information, but also understands the different intentions humans can display while collaborating with a robot. Thus, we also add a term to the loss function to be able to classify a human skeleton sequence according to the underlying intention of the human along the sequence. We have trained and validated this architecture using two distinct collaborative tasks. To achieve our objectives, we have meticulously curated two distinct datasets, showcasing human interactions with collaborative robots in real environments while performing specific tasks. Some of these tasks involve active interactions with the robot, while others involve passive interactions, where the human is in proximity to a robot. Once these datasets were created, we extracted context information for each task. Broadly speaking, context information refers to data captured within the dataset that can provide insights into the human’s goal, aiming to enhance the quality of the prediction models. The type of data encompassed within this definition varies based on the specific task at hand. For cooking tasks, contextual information might include the positions of ingredients and tools, thereby enhancing the prediction model’s understanding of the cooking process. On the other hand, for cleaning tasks, the geometry and layout of the surfaces to be cleaned could serve as relevant contextual cues. The nature of the task dictates the specific contextual elements that contribute to a more accurate and meaningful human motion prediction. For comprehensive analysis, the datasets were labeled to classify each skeleton pose with an associated human intention behind the displayed motion. The primary goal of this classification is to understand whether the human intends to collaborate in the success of the collaborative task or not.

The two human–robot collaborative tasks that we have focused on are human–robot handovers and human–robot grape harvesting in a real field. In the handover task, contextual information includes the position of the robot end effector (REE) and the location of obstacles present in the scene. On the other hand, for the harvesting task, the contextual information considered involves the position of grape bunches and the location of the box where the grapes are stored. By incorporating these contextual details, our aim is to develop more accurate and effective prediction models for human motion and intention during collaborative tasks with robots. Furthermore, we also consider the human intention during the collaboration with the robot. This human intention relates to how does the human want to interact with the robot.

Summarizing, the main contributions proposed in this work are:

- The creation of two human motion datasets for human–robot specific tasks.
- The proposal to use contextual information related to each specific task in order to increase the accuracy of the prediction model.
- The possibility to classify different human intention during the collaboration task and to modify the predicted sequence based on each specific intention class. We believe that this feature is a key element for collaborative robotics since robots should be aware of the different possible motions humans can do based on their own goals.

In the remainder of this journal, in Sect. 2 we first give a short review of the related work, in Sect. 3 we explain our 3D human motion prediction model, in Sect. 4 we describe the dataset that we created to do the validation of the model, in Sect. 5 we explain the experiments, in Sect. 6 we present the user study and finally in Sect. 7 we present the conclusions.

## Related Work

Given the variety of topics related to this work, this section will be structured in three different blocks: Human Motion Prediction, Human–Robot Handover and Human–Robot Harvesting.

### Human Motion Prediction

In this block, we will study pure human motion prediction in the classical approach, especially in the computer vision field. To do so, we propose a chronological overview of different techniques that have been recently used in the computer vision community in order to approach the human motion prediction problem.

One of the first approaches followed by the community was using recurrent neural networks (RNN), since they provided good results in other series-based problems such as text generation. In [2] by Martinez et al., the human motion prediction problem is approached as a time series algorithm, proposing a RNN architecture able to generate a predicted human motion sequence given a real 3D joint input sequence. Although the results obtained in this model are quite interesting, the work raises attention in a particular case: a non-moving skeleton can often improve results in a L2 based metric. This phenomenon has been widely used as simple baseline for many human motion prediction model proposals, such as [3–7] or [8].

Continuing along the trajectory of RNNs, a work closely aligned with our research is that of Kratzer et al. [9]. In this study, a short-term prediction RNN is employed to forecast human motion, while also considering the spatial arrangement of various objects in the vicinity of the human. Notably, objects like tables, chairs, and shelves are taken into account in the prediction process. Moreover, the work incorporates the concept of graspability for these objects, aiming to enhance the trajectory predictions for human motion. This approach demonstrates the integration of environmental factors and object interaction to further refine and optimize human motion predictions, thereby showcasing a significant relevance to our own research endeavors.

Moving on from RNNs, the community started exploring techniques leaning more towards the generative side. At this point, Variational Autoencoders (VAE) where used in order to model a latent space distribution to draw samples of sequences from said latent space. Another very interesting work is the one presented by [10], where they use Transformer Variational Autoencoder (TVAE), also using attention to predict the human motion, but they condition their prediction with the action that the human is performing, which arguably may be considered as context.

Another very popular generative model are Generative Adversarial Networks (GANs). Contextual information has been previously used in other works that deal with motion prediction. The approach from [7] is philosophically very similar to our work, since the model predictions are conditioned on the objects around the humans, such as tables or doors. The model uses a GAN architecture to exploit this added information.

More recently, the use of Graph Convolution Networks (GCN) arouse among the community, since they flexibility of the architecture allows the network to learn the dynamic dependencies of the human body. The most relevant work for our proposal is Mao et al. [11], where the temporal joint information is encoded using a discrete cosine transformation (DCT). This approach mitigates the problems related to auto-regressive models, and has yield to very good results in other works such as [12] by Aksan et al.

Moreover, in the work presented in [13] the same authors study further the convenience of working in the trajectory space rather than in the traditional pose space, allowing models to reason in longer motions rather than just being able to reason about the anthropomorphic structure of the pose.

In a similar approach, Martínez-Gonzalez et al. [14] use transformers and take advantage of the DCT conversion to tackle the prediction problem, but also include a way to allow the network to detect the activity of the human to improve the model outputs. This idea looks similar to the one presented in this work, but we fix the task and try to identify the intention of the human during said task.

The approach from [15] is also similar to [11], using graph convolutional networks and DCTs, but in this case a specific module is built in order to learn the joint relations of the body, instead of letting the attention mechanism figure out this relationships.

On a similar note, the work proposed in [16] is also able to predict the human motion while reasoning with the scene context. The first detail that looks interesting is the use of a video-game based synthetic dataset generator to create human motion data in daily scenarios (houses, offices, stairs,...). The model uses as input the RGB image of one frame and the skeleton pose history in the image 2D frame. Then, problem is approach in 3 steps, each one solved by a different network: first, the human goal is estimated to decide where the human is heading. Then, the future 2D path is generated by the following network, taking into account the scene elements that may interfere in this path. Finally, the 3D pose motion is generated along the predicted path using a transformer based network. Although the use of the scene (or contextual) information looks very promising, the data used in this work still doesn’t account for the presence of robots in the surrounding of the humans.

In recent times, the development of widely used body parametric models like SMPL [1] and the establishment of new 3D human datasets have given rise to a growing trend of approaching the human motion prediction challenge as a mesh prediction problem. A notable example can be found in the research presented by Xu et al. [17], which reflects an increasing interest in generating human predictions by considering the potential interactions that humans typically have with various objects (akin to the “context” concept applied in our work). Moreover, the suggested architecture employs a diffusion model to encode the anticipated distribution of future human-object interactions in order to make these predictions.

### Motion Prediction in Human–Robot Interaction Tasks

The field of robotics also explores predictive elements, particularly in human–robot interactions like handovers. Hoffman et al. in [18] compare anticipatory and reactive agents, highlighting the importance of predicting human intentions for smoother collaboration. Lang et al. [19] utilize Gaussian Process clustering with stochastic classification for trajectory prediction in object handovers. Additional studies on handover tasks in human–human interactions include [20] and [21]. Nemlekar et al. [22] propose an efficient method for predicting the Object Transfer Point between a robot and a human. Moreover, [23] extends the predictive approach from [11] to jointly forecast the motion of two humans during interactive tasks, leveraging past motion to learn interdependencies between their joints.

The study presented by Sung et al. [24] introduces the utilization of Gaussian Process Regression for predicting forthcoming human motions, encompassing a time frame of up to one second. Notably, the authors incorporate the concept of human intention, defined as the specific object the human intends to reach from a selection of four available positions. Moreover, this model is extended to facilitate robot planning. The robotic component of the study features a 7-degree-of-freedom manipulator, designed to execute collaborative tasks in close proximity to humans. Importantly, the model takes into account the inherent unpredictability in human behavior, underscoring the significance of robust and adaptable robot planning strategies for cooperative interactions.

### Human–Robot Harvesting

The continuous growth of the human population has heightened the importance of ensuring reliable food supplies for human sustenance. To meet the increasing demands, agricultural efficiency must be maximized. Consequently, the field of robotics has become a significant area of interest, particularly in the context of harvesting operations. Harvesting, despite appearing highly automatable, often involves delicate handling of the produce, making the study of close human–robot interactions critical.

However, recent research in the robotic harvesting field, as highlighted by [25], shows that only 6% of publications are considering human–robot interaction (HRI) strategies to tackle the operation. The majority of work has been focused on developing interfaces for human–robot interactions, with little emphasis on exploring the intricacies of such interactions.

For example, in [26], the author evaluates three different interfaces for selecting spraying areas in semi-autonomous robots, using a mouse, a Wii controller, and a digital pen. However, in these scenarios, the human user is always considered to be far away from the robot, which differs from our work, where close interactions are essential.

One interesting approach is presented in [27], where the focus is on optimizing logistic operations in the field involving various robots performing different tasks. The authors propose utilizing spatio-temporal information of human activities within a Hidden Markov Model to predict the whereabouts of human pickers at any given time. Based on this prediction, a multi-robot logistics model is employed to schedule the robot fleet, ensuring that the robots can anticipate the human needs. As a result, they achieve an 80% reduction in unproductive time compared to a fully manual operation.

In another approach [28], addresses emergency situations during farmers’ work. Although no robotic systems are involved in this study, the researchers consider the workers’ poses. The poses are captured using wearable devices on the workers and are used to train a classifier capable of identifying the farmer’s activity and detecting potential risks.

Our method diverges from all of the aforementioned approaches in two significant aspects:

- Our model is trained on a custom dataset where interaction occurs between a real robot and humans, resulting in highly natural and realistic human reactions and movements.
- Our model is trained in such a manner that it can leverage an understanding of the intentions behind human actions during interaction by discerning hidden information cues from body poses. This knowledge is subsequently utilized to enhance the prediction of motion sequences..

**Fig. 2:**
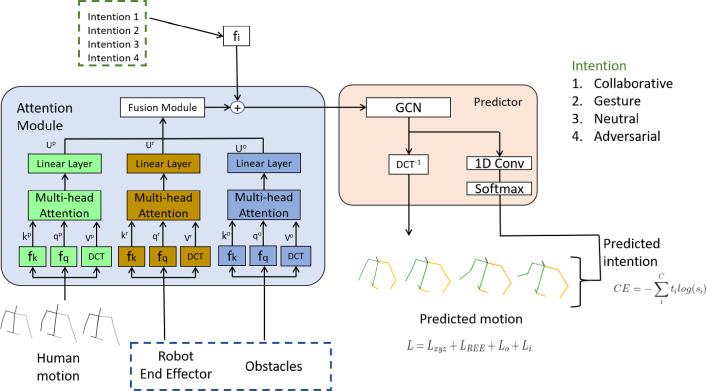
Model overview for the specific case of the HRI handover. The model has three inputs, the human motion sequence seen in the past, and the context related to the specific task: REE and obstacle position. In the case of the harvesting operation, instead of REE and obstacles we only input the bunches position. This input can be changed depending on the specific task. The model allows to add as many attention channels as desired. To finish the encoding process, each attention output is fused in the fusion module. Additionally, the desired intention can be added in the fusion module in case that a specific type of output sequence must be produced. At this point, the decoding process starts, using a graph convolution network (GCN) to generate the predicted motion of each joint in the frequency domain. This output is processed in an inverse discrete cosine transform (IDCT) to obtain the predicted human motion in cartesian coordinates. Paralelly, the predicted joint motion is also processed with a one dimensional convolution neural network able to classify each predicted frame into one of the defined intentions

## Model

We propose a modified deep learning model with attention, based on Mao et al. [11], which can predict both future 3D human motion and intention (see Fig. 2).

### Problem Definition

Given a motion history encoding of human motion, $X_{1:N}^{p} = [x_1^{p}, x_2^{p}, x_3^{p}, \ldots , x_N^{p}]$ where $x_i \in \mathbb {R}^K$ (3D coordinates of each joint), the objective is to predict T future poses $X_{N+1:N+T}^{p}$ and human intention for each frame.

Additionally, to incorporate context information relevant to the task, we introduce a general context queue, $X_{1:N}^{c}$, containing temporal or fixed information related to the given task.

For the handover case, the focus is on the REE as the objective is to place the object near the end effector. Hence, we introduce a new queue, $X_{1:N}^{r}$, which encodes the REE’s 3D motion history as $[x_1^{r}, x_2^{r}, x_3^{r},\ldots , x_N^{r}]$, where each $x_i^{r}$ is a 3D vector in $\mathbb {R}^3$.

Furthermore, the context information for the handover task includes the 3D positions of obstacles in the scenario. We encode the obstacle positions as $X_{1:N}^{o} {=} [x_1^{o}, x_2^{o}, x_3^{o},\ldots , x_N^{o}]$, where each $x_i^{o}$ contains the 3D coordinates of three obstacles, represented as the obstacle centroids, and belongs to $\mathbb {R}^{3 \times 3}$.

On the other hand, for the harvest operation context, we consider the positions of both the grape bunches and the storage box as goals (each one is the goal during a certain sub-task of the operation, the bunches are the goal during the "harvest" itself, and the box is the goal during the "drop" phase). Thus, $X_{1:N}^{g}$ represents the position of the goals relative to the robot in each frame, with each $x_i^{g}$ belonging to $\mathbb {R}^3$.

In both operations, we define a goal intention *i* with $i \in [0, c-1]$ and $i \in \mathbb {N}$, where *c* is the number of intention classes (refer to Sect. 4 for further information). This value determines the intention that the human will express in the predicted frames $\hat{i}_{N+1:N+T}$.

### Architecture

#### Attention Channels

The first change over the original architecture from [11] involves the addition of multiple input channels. Unlike the original model that only considered human 3D skeleton data, we now aim to include multiple context information. For this purpose, we introduce an attention channel for each context queue under consideration.

To compute attention scores, we divide each input sequence $X_{1:N}^{p}, X_{1:N}^{r}, X_{1:N}^{o}$ into $N - M - T + 1$ sub-sequences ${X_{i:i+M+T-1} ^{j}}^{N-M-T+1}_{i=1}$, where *i* is the time-step index of the sub-sequence, and *j* refers to the information channel (*p*, *r*, or *o*). This division ensures that each sub-sequence has $M+T$ frames. During training, the aim will be to predict the *T* frames based on the *M* previous ones for each sub-sequence. N is the number of frames forming the input sequence, M is a sequence of frames included inside the Nth long input sequence and T is the sequence of frames following the mentioned M long sequence. Following the idea that every M long sequence has a corresponding T long sequence of "future" frames, we can train a network able to predict the future T frames of the N long sequence by looking at the last M frames of the N long sequence.

By creating this data structure, we can define the classical attention formulation of *keys*, *values*, and *query*. We consider all possible *M*-length segments of the sub-sequence $X_{i:i+M-1}^{j}$ as *keys* (length M). The entire sub-sequence $X_{i:i+M+T-1}^{j}$ is transformed into the frequency domain using a discrete cosine transform (DCT), which produces the *value* (length M+T) for each *key*. Finally, the last *M* frames of the sub-sequence $X_{N-M+1:N}^{j}$ serve as the *query* (length M), leading the network to output the next predicted T frames after the query.

The keys and query undergo processing by mapping functions $f_k^{j}$ and $f_q^{j}$, respectively, before computing attention scores. These functions encode the input data into vectors of size $\mathbb {R}^d$ using neural networks comprising three 1D convolution layers, followed by batch normalization and ReLU activation.

1 $$\begin{aligned} k_{i}^{j} = f_{k}^{j} \left( X_{i:i+M-1}^{j} \right) , q^{j} = f_q^{j} \left( X_{N-M+1:N}^{j} \right) \end{aligned}$$

Computing attention scores involves multi-head attention, as described in [29]. This entails performing the attention operation in parallel for each defined head. Each head takes a distinct embedding, $k_{i}^{h, j}$ and $q^{h, j}$, for each head *h* in the range [1, *H*]. Finally, attention scores for each channel and head are calculated.

2 $$\begin{aligned} a_{i}^{h, j} = \frac{q^{h, j} k_{i}^{h, j^{T}}}{\sum _{i=1}^{N-M-T+1} q^{h, j} k_{i}^{h, j^{T}}} \end{aligned}$$

#### Information Fusion

Each attention channel output $U^{h, j}$ is calculated by multiplying its attention scores $a_{i}^{h, j}$ with the corresponding "value" $V_{i}^{h, j}$ of the given "key":

3 $$\begin{aligned} U^{h,j} = \sum _{i=1}^{N-M-T+1} a_{i}^{h, j} V_{i}^{h, j} \end{aligned}$$

Each $U^{h, j}$ is in $\mathbb {R}^{K \times (M+T)}$. The outputs from all heads are concatenated and then input into a linear function $f_h$.

4 $$\begin{aligned} U^{j} = f_h \left( U^{1, j} \mathbin \Vert U^{2, j} \mathbin \Vert \cdots \mathbin \Vert U^{H, j} \right) \end{aligned}$$

Having transformed each input sequence into an embedded tensor $U^{j}$, we proceed to aggregate these tensors into a unified tensor *U* through a weighted sum operation. Notably, the model itself learns these weights through training. This approach is rooted in the concept of enabling the model to determine the relative significance of each input sequence, thus facilitating dynamic consideration of inputs based on their relevance.

We compute the weighted sum of all attention channel outputs by multiplying each output by a learned weight $\alpha ^j$:

5 $$\begin{aligned} U = \alpha ^{p}U^{p} + \alpha ^{r}U^{r} + \alpha ^{o}U^{o} \end{aligned}$$

#### Intention Conditioning

The output *U* is merged with the desired intention conditioning module, using the desired human intention represented by *i*. This desired intention represents the intention that we want to display in the output, allowing us to obtain different output motions depending on the selected intention. This intended intention should not be conflated with the predicted intention of the human. The intention conditioning offers an elective capability of the model, enabling it to guide the forecast sequences in a specific direction. This can prove valuable, such as generating diverse motions corresponding to the specified intentions, equipping the robot to anticipate various potential scenarios. In scenarios where this functionality is not utilized and no specific intention is imposed, it remains advantageous to not solely predict forthcoming motion but also to classify it into one of the intention categories, empowering the robot to respond appropriately. That is the goal of the predicted intention.

A function $f_i: \mathbb {N} \rightarrow \mathbb {R}^{K \times (M+T)}$ maps the intention information. This function consists on a two layered neural network, each layer consisting on a linear layer with a ReLU activation function separating between the two layers and no activation function after the second layer to use the output directly as an embedding of the intention:

6 $$\begin{aligned} U' = U + i', \quad i' = f_{i}(i) \end{aligned}$$

#### Motion and Intention Prediction

Output $U'$ is utilized by the Graph Convolutional Network (GCN) to reconstruct the predicted skeleton motion $\hat{X}{N+1:N+T}$ in a similar manner as presented in [11]. Additionally, an additional output from the GCN is the predicted human intention for each frame $\hat{i}{N+1:N+T}$, which is generated using extra layers at the GCN’s end. These layers consist of two 1D convolution layers with a ReLU activation function in-between. Subsequently, a Softmax layer is applied to solve a multi-class classification problem for each frame, producing the predicted human intention.

#### Loss Function

To optimize the model and generate realistic human motions, several loss terms are employed. The primary component is the $L_2$ distance between the predicted motion joint positions and the ground truth, denoted as $L_{xyz}$.

Moreover, predicting the human’s intention in each frame is always a goal. To address this multi-class classification problem, we utilize a cross-entropy loss term $L_i$. The cross-entropy loss function allows the model to make accurate predictions of human intention.

For the handover case, we take specific measures to enhance the quality of predictions. Firstly, we incorporate a term $L_{REE}$. This term involves the $L_2$ distance between the human’s right hand and the REE. By discouraging predictions where the human’s hand is too far from the REE during the handover, we promote more realistic and plausible predictions.

Furthermore, to ensure that the predictions do not allow the human to cross any obstacles in the scenario during the handover, we introduce a loss term called $L_o$ in the overall loss calculation. The purpose of this term is to apply a significant penalty on predictions where the human’s hips cross any obstacles. This measure helps to maintain safe and obstacle-free interactions during the handover task.

7 $$\begin{aligned} L = L_{xyz} + L_{REE} + L_o + L_i \end{aligned}$$

In the case of the harvest operation, we add a term $L_g$ in the loss function. Similar to the REE term in the handover case, $L_g$ also serves as a penalty to discourage output sequences where the human’s right hand doesn’t reach the goal (grape bunch or box). The penalty is based on the $L_2$ distance between the human end effector (HEE) and the specific goal (bunch or box). This measure aims to promote predictions where the human successfully reaches the designated goal during the harvest operation, leading to more accurate and task-compliant predictions.

8 $$\begin{aligned} L = L_{xyz} + L_g + L_i \end{aligned}$$

No weights were used to prioritize one loss over the rest.

**Fig. 3:**
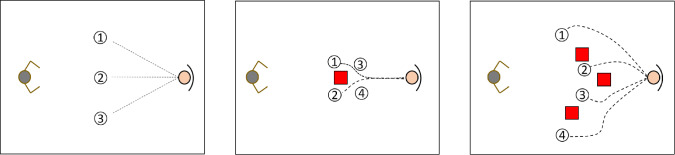
Each subfigure illustrates a top perspective of the handover sequence for the three possible scenarios. In the first scenario (left), the robot is positioned on the left side, and the human is on the right side, with no obstacles around. The three lines emanating from the human indicate the three different paths to the locations where the robot will receive the object. In the second scenario (middle), there is one obstacle (represented by a red square) between the robot and the human. In this case, four different locations were considered as delivery spots. Lastly, the third scenario (right) involves the handover operation occurring with three obstacles present in the scene. Similar to the second scenario, there are four locations where the robot is expected to receive the object. It is important to note that in all of these scenarios, the spots where the handover takes place are predetermined. During the recording, the delivery spot is sent to the robot planner, enabling the robot to navigate to the destination

## Datasets

We created two datasets in a similar fashion, the Robot Handover Dataset and the Harvest Dataset. The data collection of the handover was approved by the ethics committee from the Polytechnic University from Catalonia, certificate number 2022.1.

In this study, we have categorized human intentions into several distinct classes for the purpose of analyzing human–robot collaborative tasks. Specifically, we have defined four primary intentions: collaborative, gesture, neutral, and adversarial. These intentions capture various scenarios and interactions between humans and robots.

In the context of the handover task, the following intentions are considered: Collaborative intention, where the human approaches the robot to deliver an object, carries out the handover, and then moves the hand away from the robot’s hand. Neutral intention, where the human intends to continue the collaborative task but exhibits a passive behavior, not raising the hand when the actual handover should take place. Adversarial intention, where the human avoids the handover task by moving away from the robot. Gesture intention, which occurs when the human performs any motion not covered by the previously mentioned intentions, such as checking his smartphone or waving to the robot.

Similarly, we can decompose the human intentions in the harvesting operation. The human follows a collaborative intention when it harvests the grapes or approaches the robot with a cluster of grapes to deliver them in a box carried by the robot. The human follows a adversarial intention, when for example, it moves away from the robot after collecting the grapes instead of delivering them in the robot box. Again, the human shows a gesture intention when, at any given time, the human makes a motion different to the motions previously described. The neutral intention wasn’t considered in this specific case because it was hard to define since the robot was not moving during the data collection for security reasons.

These intention classifications offer a structured framework for analyzing and understanding human behaviors in the context of collaborative tasks, enhancing the overall comprehension of human–robot interactions.

### Robot Handover Dataset

Ten volunteers, consisting of 5 women and 5 men, with ages ranging from 25 to 60, participated in the recordings. Each volunteer recorded all scenarios, resulting in 33 sequences per volunteer and a total of 330 sequences in the dataset. The duration of each sequence varies between 4 and 15 s. The human and robot start each sequence 6 ms apart.

In our previous work [30], we established a custom dataset in our laboratory. However, for this study, we wanted to investigate the impact of obstacles in the environment and the differences when the human is the one handing over an object. To achieve this objective, we created a new dataset with revised conditions.

The dataset was generated by involving the anthropomorphic robot, IVO [31], and human volunteers to perform a handover task. In this scenario, the human served as the *giver*, while the robot acted as the *receiver* (as illustrated in Fig. 3). The human played the role of *leader*, and the robot was the *follower*, with the robot required to mimic and follow the human’s movements to reach the object. During the experiment, both the human and the robot navigated around obstacles and extended their arms to reach each other. At the end of each sequence, the human passed a 10 cm cylindrical object to the robot’s end effector using their right arm, and the robot grasped the object.

The robot’s behavior was preprogrammed to navigate towards distinct predetermined waypoints, strategically chosen to encompass a range of relevant interaction scenarios. In each sequence, explicit instructions were provided to the human participant regarding their intended destination. Meanwhile, the robot was guided using a standard planner to approach a location in proximity to the human’s designated goal position. See Fig. 3 for a visual representation of the used setup.

**Fig. 4:**
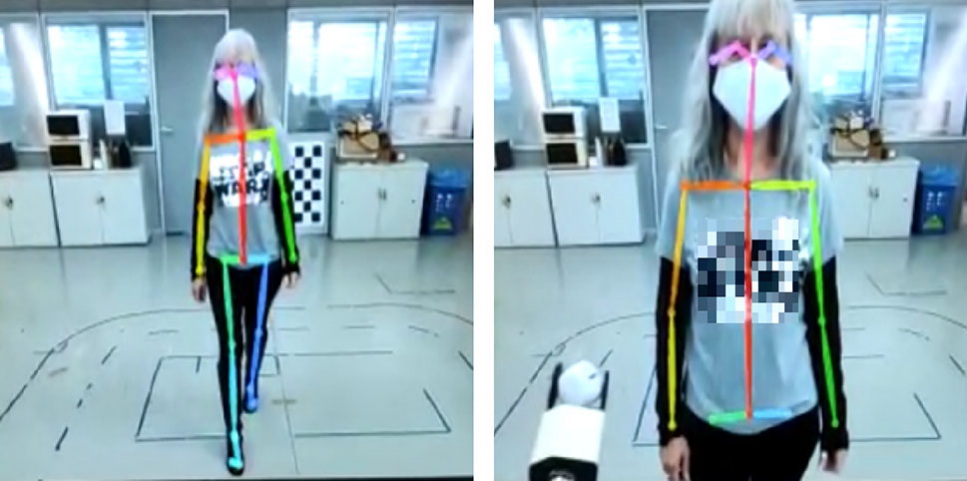
Sample sequence of the human–robot handover. The human skeleton is extracted using a pose estimator. Then, the 3D location of each joint is reconstructed using the depth map of the robot RGBD camera

The Intel RealSense D534i camera located inside the robot’s head recorded a video of each sequence at 10 frames per second (fps). The recording stopped when the human placed an object in the REE. The human’s skeleton was extracted from each video using Mediapipe [32] to determine 2D joint locations in the image. These 2D joint positions, along with camera depth map data, were then used to calculate the 3D joint coordinates relative to the robot base. Only the upper body of the human, from the hips to the head, was utilized to avoid leg occlusions during close proximity to the robot. In Fig. 4 for an example of the robot point of view.

In three different scenarios, the volunteers delivered a cylindrical object to the robot. The first scenario had no obstacles, the second scenario had one obstacle, and the third scenario featured three obstacles. To capture all the possible approaches a human could take to reach the robot, different approaching paths were defined. For the first scenario, three paths were designated, four for the second scenario, and another four for the third scenario. The goal was to examine two factors: how the model responds to human lateral movement (previous work only considered straight trajectories) and how obstacles impact predictions. The obstacles used were cardboard boxes, measuring $20 \times 20$ cm at the base and 50 cm in height. The object’s position was determined through trial and error to create challenging situations for both the robot and human. The object’s position was not tracked by the robot and was assumed to be in a known location.

During the data collection, the human volunteers were asked to repeat each trajectory three times. First, they were asked to perform the task naturally, following the leader-follower behavior, as expected. Second, they were asked to perform a random gesture during the task while still delivering the object as expected. This approach served two primary purposes: firstly, to capture poses that deviate from the typical distribution observed during a handover operation, and secondly, to accommodate the potential occurrence of human gestures that are unrelated to the specific task within a real handover scenario. Finally, the users were asked to walk towards the robot and then deliberately not deliver the object (adversarial behavior). These different behaviors were included to study the impact of human intentions on motion prediction. After recording all the sequences, a visual inspection was performed for data validation. Additionally, each recorded frame was labeled with an intention class: Collaboration, Gesture, or No collaboration. This labeling allowed for further analysis of the human intentions during the handover task.

- Collaboration: the human is willing to deliver the object to the robot.
- Gesture: the human is performing a gesture (we do not differentiate between communicative and non-communicative gestures).
- Neutral: the human does not raise the right hand towards the robot, but will not make any movement to oppose the robot.
- Adversarial: the human moves the right hand away from the robot.

**Fig. 5:**
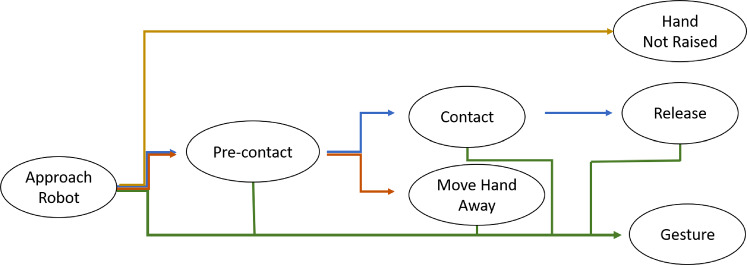
This figure represent the different phases and how the intention is defined in the handover task. Each node in the graph represents a phase of the harvest task. The blue arrow path represents the phase order followed by the user when he/she is collaborating. The orange arrow represents the path followed when the human is not collaborating in a neutral way. The red arrow represents the phase order for the adversarial intention. The green arrow represents all the possible paths for the gesture intention, which can happen after every single phase

The relation between phases and intention can be seen in Fig. 5

The REE position and robot odometry were recorded during all sequences.

### Harvest Dataset

We created a second dataset to study a more complex task: grape harvesting. This task involves one or more humans collecting grapes while a robot supports them in the area. The grape variety considered in this task is sold as table fruit, and it is essential to minimize imperfections derived from the harvesting to ensure the grapes look as good as possible. Thus, a manual operation is preferred.

The dataset comprises 10 participants, consisting of 9 men and 1 woman, aged between 24 and 60 years old. Each participant performed the grape harvesting operation three times. In the first scenario, the participants harvested the grape bunches and dropped them into the box carried by the robot, demonstrating a collaborative intention. In the second scenario, participants performed gestures related to the harvesting activity, such as drinking water, wiping sweat from the forehead, etc., representing a gesture intention. Finally, in the third scenario, participants harvested the grape bunches and then moved away from the robot while carrying the bunches, reflecting an adversarial intention. Consequently, the dataset consists of 30 sequences in total, ranging from 15 to 40 s in duration.

Typically, in a non robotic way, the task can be described by the following phases:

1. The human, using scissors, grab the grape peduncle with the thumb and the index fingers. Then, with the other hand, the human cuts the peduncle.
2. The human leaves gently the peduncle in a box placed nearby.
3. Steps 1 and 2 are repeated until the surrounding area is cleared of grape bunches.
4. If the box is full of grape bunches, the human moves the box to a storage point where other boxes are stored. Otherwise, the human moves the box to the next harvesting area.
5. The human moves to the next harvesting area, and repeats all the operations until all the grape bunches are collected.

The way phases and intention interact is schematized in Fig. 7.

We aim to explore the human–robot collaboration aspect of the task. For this purpose, we utilize a TIAGO robot with a modified base platform. The robot’s wheels have been replaced by caterpillar tracks to allow maneuvering in the field effectively. Additionally, the base has been designed to accommodate a box, which is essential for the collaborative task.

The interaction we are studying involves enabling the robot to follow the human carrying the box and managing all the box replacement operations.

**Fig. 6:**
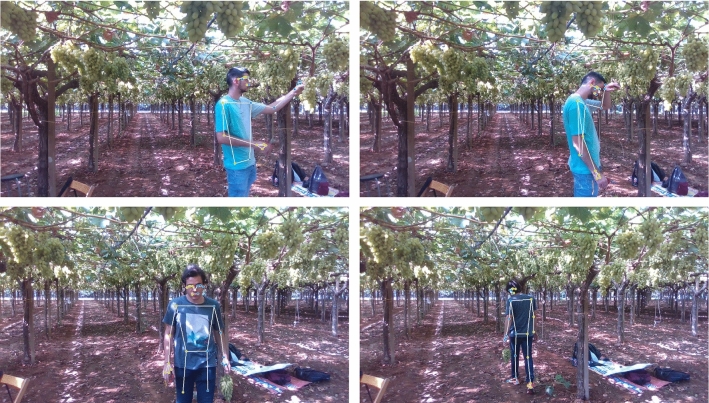
Sample frames from the dataset. Top-left: The user is in the "harvest" phase. Top-right: The user is in the "gesture" phase. Bottom-left: The user is in the "approach box" phase. Top-right: The user is in the "move away" phase. All this images are recorded from the robot point of view

Given that the robot will be operating in a harvesting field surrounded by multiple humans, it is crucial for the robot to predict how people will move to avoid any dangerous movements. Moreover, the robot needs to understand what each human is doing to provide timely assistance when necessary. Figure 6 illustrates some samples of the collected data, showcasing the complexity and diversity of the scenarios in the harvesting field.

**Fig. 7:**
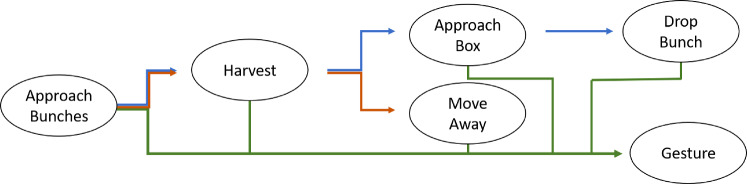
This figure represent the different phases and how the intention is defined in the harvest task. Each node in the graph represents a phase of the harvest task. The blue arrow path represents the phase order followed by the user when he is collaborating. The red arrow represents the phase order for the adversarial intention. The green arrow represents all the possible paths for the gesture intention, which can happen after every single phase

An important factor to consider is that, as mentioned earlier, the robot remained stationary during the data collection process due to the non-operational navigation tools at the time. In subsequent data collection efforts, the robot’s motion will be integrated to allow for a comprehensive analysis of its impact on the collaborative interactions. This future data collection phase will provide valuable insights into how the robot’s movements influence and shape human–robot collaboration dynamics.

## Experiments

The experiments will be presented in two different sections: the first describing the experiments with the Robot Handover Dataset and the second describing the experiments with the Harvest Dataset.

**Table 1:** Results obtained across the handover validation dataset

Model	Body $L_2$ (m)				$\le$ 0.35 m (%)	$\le$ 0.40 m (%)	Right hand $L_2$ (m)				Intention accuracy (%)
Seconds	1	1.5	2	2.5	2.5	2.5	1	1.5	2	2.5	2.5
RNN [2]	0.411	0.723	0.745	0.793	3.49	11.62	0.349	0.606	0.847	0.677	–
$\Box$ REE	0.208	0.361	0.504	0.403	34.13	37.14	0.099	0.171	0.235	0.188	–
$\Box$ obstacles											
$\Box$ intention											
REE	0.195	0.338	0.476	0.378	**41**.**65**	45.78	0.092	0.151	0.164	0.174	56.45
$\Box$ obstacles											
$\Box$ intention											
$\Box$ REE	0.210	0.398	0.401	0.444	41.31	44.87	0.097	0.169	0.234	0.187	62.02
obstacles											
$\Box$ intention											
$\Box$ REE	0.237	0.405	0.411	0.453	30.81	36.15	0.088	0.155	0.173	0.173	86.29
$\Box$ obstacles											
intention											
REE	0.199	0.341	0.476	0.381	41.60	**47**.**38**	0.089	0.154	0.215	0.172	74.16
obstacles											
$\Box$ intention											
REE	0.196	0.331	**0**.**367**	0.375	34.84	38.86	0.083	0.143	0.203	0.162	85.44
$\Box$ obstacles											
intention											
$\Box$ REE	0.199	0.347	0.392	0.387	40.22	43.73	0.084	0.150	0.163	0.170	88.69
obstacles											
intention											
REE	**0**.**183**	**0**.**321**	0.444	**0**.**355**	32.15	35.73	**0**.**078**	**0**.**135**	**0**.**139**	**0**.**151**	**88**.**90**
obstacles											
intention											

The table contains the results of testing different models, with each row representing a specific model. Here is a description of each column: Model: The specific model being tested in each row. Body L2: The mean L2 error between the predicted joint positions and the ground truth for the whole human body, averaged over the entire sequence, also known as mean per joint position error (MPJPE). This metric has been analyzed in four different time horizons: one second, one second and a half, two seconds and two seconds and a half. $\le 0.35$ m: The percentage of frames in the sequence where all the predicted joint errors are below or equal 0.35 meters. This metric hasn’t been analyzed in different horizons because the 0.35 m threshold was selected considering the 2.5 s horizon, hence the metric would be 100% for almost every time windows except for the last one. $\le 0.40$ m: The percentage of frames in the sequence where all the predicted joint errors are below or equal 0.40 m. The same comment in the previous metric about the time windows can be made in this case. Right Hand L2: The mean L2 error between the predicted joint positions and the ground truth just for the right human hand, averaged over the entire sequence. This metric has also been analyzed in four different time horizons: one second, one second and a half, two seconds and two seconds and a half. Intention Accuracy: The percentage of poses where the intention was correctly classified by the model. These metrics are used to evaluate the performance of each model in predicting human motion and intentions. In this case the time windows cannot be applied since the network in charge of the intention estimation looks at the whole predicted sequence by design. Lower L2 errors and higher percentages of frames with low errors indicate more accurate predictions, while a higher percentage of correctly classified intentions indicates better intention prediction by the model. The bold numbers mark the best results for each column metric. Note that the second column, where no checkboxes are checked, corresponds to the backbone model from [11]

### Handover Experiments

#### Training Details

The data split between training and validation was set to 85 during training, we utilized 50 frames as input, corresponding to 5 s of recording, and produced output of 25 frames, equivalent to 2.5 s of predictions. We kept the number of attention heads fixed at 10 and employed the Adam optimizer for training. Additionally, we performed an ablation study to assess the impact of individual features of the model, such as the number of attention heads, attention channels, and intention conditioning.

To ensure fair comparison, we trained and validated the original non-modified human motion prediction model on our dataset. However, it is crucial to note that since we evaluated this model on our own dataset, the results obtained might differ from the results reported in the respective paper, where they typically trained their models on larger datasets like H3.6M [33] and AMASS [34].

The results presented in Table 1 are obtained from our validation dataset and offer a comprehensive overview of the performance of different models on our dataset. Since the amount of data collected in the dataset is relatively small, the results were obtained using the leave-one-out technique, where we trained first the model using the subject one as the testing data, trained the model with the rest of subjects, and evaluated it with subject one. Then, subject one was incorporated into the training dataset, and subject two was used as the evaluation subject. This process was repeated for all the subjects, and the evaluation metrics were averaged for all the subjects.

The technique used to train the models was early-stopping, checking the train and validation loss curves and stopping the training model once the validation loss was clearly overfitting. The number of epochs used was between 700 and 1000 epochs, depending on the model. The learning rate used was a decaying learning rate, starting from a 0.001 value. The optimizer used was the Adam optimizer.

During training and testing, when the intention conditioning module was used, we input the intention seen in the ground truth sequence.

#### 3D Human Motion Prediction Experiments

In our evaluation process, we determine the accuracy of our model by computing the $L_2$ distance between our predicted sequences and the ground truth sequences in Cartesian coordinates for each input sequence. The resulting errors for each sequence are recorded and can be found in Table 1. These errors represent the amount of deviation from the actual human motion.

In addition to computing the $L_2$ error, we also calculate the number of frames within each sequence that have an error less than or equal to 0.35 m and 0.40 m. The percentage of successful frames provides insight into the overall accuracy of the model.

Another crucial aspect of our evaluation is the focus on the accuracy of the right hand of the human, referred to as the HEE. This is particularly important because the right hand is the primary body part involved in the handover task.

To summarize, the most significant metrics for evaluating the performance of the model include the full body and right hand $L_2$ accuracy, as well as the number of frames with errors below 0.35 m and 0.40 m. These metrics provide a comprehensive understanding of the model’s accuracy and allow us to determine the quality of the predictions.

As observed from the results presented in Table 1, incorporating context into the predictions has a significant positive impact on the accuracy of the model. The inclusion of the REE position information leads to a notable reduction in the error of the right hand, which is critical in the handover task. Moreover, the addition of REE information improves the accuracy of the entire upper body, possibly due to the improved spatial relationship between joints.

Additionally, including the position of obstacles appears to reduce the number of frames with errors greater than 35 cm and 40 cm. This effect may be attributed to the fact that the skeleton is less likely to follow impractical paths, resulting in trajectories that align more closely with the ground truth.

The introduction of intention conditioning into the model significantly enhances the accuracy of predicted intentions. However, it is essential to interpret these results cautiously. By providing the model with information about the intended intention from the ground truth sequence, the improvement in accuracy could be misleading.

**Fig. 8:**
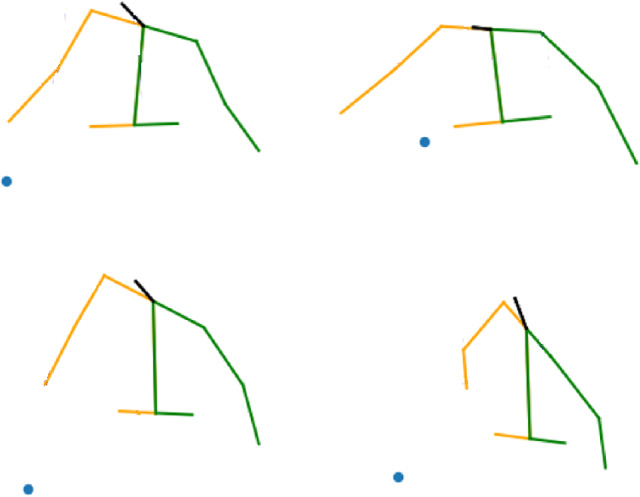
Last frame of the predicted sequence generating different intentions from the same input sequence.. The blue dot corresponds to the REE. Top-left: Collaborative. Top-right: Gesture. Bottom-left: Neutral. Bottom-right: Adversarial

Thus, although conditioning with the desired intention does not boost significantly the $L_2$ based metrics, this conditioning allows the model to generate different trajectories based on the distribution associated to each specific intention class (as depicted in Fig. 8). Even when presented with the same input sequence, the model can produce multiple predicted motions, each corresponding to a different human intention. This capability makes the model more adaptable and capable of understanding and responding to different intentions, thus potentially contributing to improved human–robot collaboration in the handover task, even though the joint use of these different predictions is not explored further in this work.

#### Handover Human–Robot Validation

The predictor model was successfully integrated into the robot’s system. The model was encapsulated as a ROS node, and the forward calculation was performed on an NVidia Jetson Xavier platform located inside the robot. For each new message received from the skeleton extractor, the model generated 25 frames of future predictions, representing the next 2.5 s. The main goal of this integration was to evaluate the feasibility of the predictor in real-time use with the robot, rather than focusing on specific accuracy measurements.

The testing phase with two volunteers who were not part of the dataset collection yielded encouraging results. For the intention conditioning, a cooperative approach from the human was assumed, but future work will explore the utilization of the model’s predicted intention for the next time step prediction.

To achieve real-time performance, some model parameters had to be adjusted. The REE was used to condition the predictions, and the scenario assumed no obstacles.

Since the human intention cannot be known in advance, the intention conditioning was set to “cooperate.” However, a closed-loop system could be implemented by training an intention classifier that can read the input data and determine the human’s current intention. This aspect goes beyond the scope of our current work.

During the testing, the model was evaluated while the volunteers approached the robot from different angles. The results demonstrated that the predictions were consistent with the human’s trajectory, with the human consistently facing the robot in the final stages of the interaction.

Overall, the real-time implementation of the model on the robot showcases its potential practicality and effectiveness in human–robot collaborative scenarios, such as the handover task.

#### User Study

The results from the previous section indicate that the robot can accurately predict and deliver an object to a person. To assess the impact of the prediction module on the usability and comfort of the robot from the user’s perspective, a user study was conducted. The study aimed to test the hypothesis that participants would perceive an improvement in the robot’s intelligence and usability when using the prediction module compared to not using it.

Fifteen participants (8 men and 7 women) between the ages of 19 and 50 were selected for the experiment, representing various university majors and occupations, including computer science, mathematics, biology, finance, and chemistry. The participants in the study had varying degrees of experience with robots, ranging from individuals who work with robots on a daily basis to those who had never seen a humanoid robot before. Each participant was randomly assigned to either have the prediction module activated or not during the delivery of an object. The order of the experiment was randomly assigned at the beginning of the study. This means that some participants started the experiment with the robot following their current position, while others began with the robot following their predicted position. The random assignment was done to eliminate any potential bias or order effects that could influence the participants’ responses during the study. Certainly, during the study, participants were intentionally kept unaware of whether the robot was utilizing the motion prediction module. This blind setup ensured that the participants’ interactions with the robot were unbiased and unaffected by any prior knowledge of the robot’s capabilities. Also, each participant engaged in approximately 5 min of collaboration with the robot, and the intentionally short duration prevented participants from becoming accustomed to the robot’s behavior over an extended period of time. This approach aimed to maintain the authenticity of participants’ responses and perceptions throughout the study.

To focus solely on the effect of the prediction information on the handover task, the model was tested without any interference from the REE or obstacle positions, and obstacles were not present in the environment. The model was conditioned with the assumption of a collaborative human intention, since we instructed the users to always deliver the object to the robot.

**Fig. 9:**
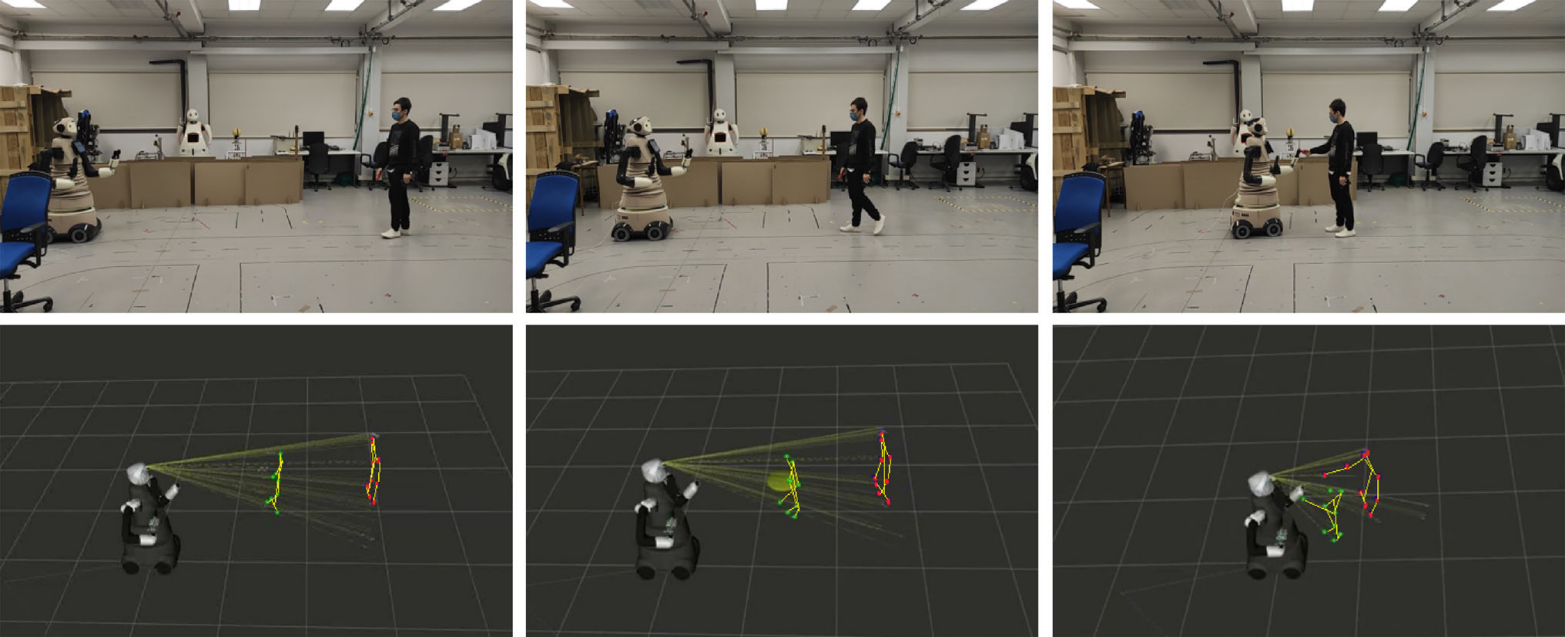
Top: Representation of the user study task. The human walks towards the robot to deliver the object. Bottom: Visualization in ROS of the future prediction of the human motion (green dots) and the current position of the human (red dots), together with the robot model. The robot could follow either the predicted points or the current points

The procedure for the user study was as follows:

1. The human motion prediction network was integrated into a ROS node and installed on an NVIDIA Jetson Xavier, which was connected to the robot. The network used during this study was not conditioned with the REE or the obstacle’s position.
2. It was randomly determined whether the first run of the experiment would use the prediction or not.
  - If the prediction was used, the robot’s navigation goal was defined as the predicted position of the right human wrist 2.5 s in the future. The model continuously recalculated this goal every 125 milliseconds based on the latest 50 poses captured by the robot camera.
  - If the prediction was not used, the robot’s navigation goal was set to the current position of the right human wrist observed by the robot camera.
3. The robot and the human start the operation standing 6 m away from each other.
4. As the robot and human approached each other, the robot would stop and raise the REE when it came within 1.5 ms of the human, indicating its readiness to take the object.
5. This experiment would be repeated three times.
6. After the interaction, the user was asked to fill out a poll rating the quality of the interaction.
7. If the first run of the experiment used the prediction information, steps 3, 4, and 5 were repeated without using the prediction information. Conversely, if the first run did not use the prediction information, steps 3, 4, and 5 were repeated using the prediction information.

A representation of the experiment sequence can be seen in Fig. 9.

After the task, the participants completed a questionnaire based on [35, 36] that assessed their perceptions of the robot’s sociability, naturalness, security, and comfort. The questionnaire was designed based on [37]. The independent variables considered were whether the robot predicted the human’s intention or not, while the main dependent variables were the participants’ perceptions of the aforementioned characteristics.

**Fig. 10:**
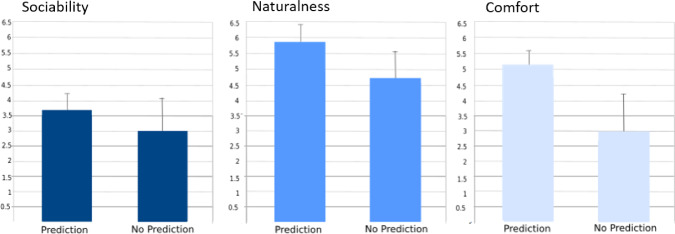
Results obtained from the user study. The results show that the use of the prediction increase the feeling of naturalness, security and comfort during the interaction with the robot

To analyze the results, the survey questions were grouped into three scales: sociability, naturalness, and comfort. Both scales had a reliability score above the commonly used 0.7 level, as determined by the Cronbach’s alpha test. The scale responses were computed by averaging the results of the survey questions in each scale. ANOVAs were conducted on each scale to determine any differences between the two robot behaviors. The Shapiro-Wilk test was used to verify that the dependent variable was normally distributed.

The results of comparing the two behaviors are depicted in Fig. 10. The pairwise comparison with Bonferroni showed no significant difference between the two behaviors for sociability, with $p=0.3$ and $p=0.17$, respectively. However, a significant difference was observed for both naturalness and comfort, with $p<0.05$ in both cases.

In conclusion, the results suggest that if the robot is able to predict the human’s intention, the acceptability of the robot will increase. The participants perceived the robot to be more natural and comfortable when it could predict their intentions during the handover task.

### Harvesting Experiments

#### Training Details

The split between training and validation data is 85% for training and 15% for validation.

Training the model with a smaller dataset compared to widely used human motion datasets like H3.6M [33] and AMASS [34] presents a challenge in achieving robust predictions. To address this, we adopted two different approaches.

Firstly, we applied data augmentation using noise in the sequences, which helped to create variations in the dataset and improve generalization. The noise was introduced in two ways:

- Random Joint Noise: We added small Gaussian noise $\mathcal {N}(0.03, 0)$ to each human skeleton joint position with a probability of 0.5 for each joint. This perturbation slightly modified the sequences without altering the anthropomorphic shape of the skeleton.
- Random Translation: We applied a random horizontal translation $\mathcal {N}(0, 2)$ to the entire sequence, except for the coordinate corresponding to the human height. This introduced variability in the dataset, simulating the harvest sequences occurring at different distances.

The second approach involved adjusting the model architecture to reduce the number of learnable parameters on each layer while increasing the number of hidden layers. By doing so, we aimed to decrease the model’s dependency on large amounts of data and allow it to capture more complex relationships.

Despite the smaller dataset, the input and output sequence lengths remained unchanged, with the input sequence consisting of 50 frames (5 s) and the output sequence consisting of 25 frames (2.5 s). The number of attention heads was still set to 10, maintaining consistency with the handover experiments.

By combining data augmentation, architectural adjustments, and the other strategies mentioned earlier, we were able to improve the model’s performance and achieve accurate predictions on the validation dataset. The final model demonstrated its potential in human–robot collaboration tasks, making predictions based on context information and human intention.

Similarly to the handover operation, during training and testing of the model, if the intention conditioning module was used, we input the intention seen in the ground truth sequence. The training technique, learning rate, optimizer and other details was the same as the handover experiments, the only difference being that generally the models started overfitting earlier, around 500 epochs.

**Table 2:** Results obtained across the harvesting validation dataset. The meaning of the title columns are the same as in Table 1

Model	Body $L_2$ (m)				$\le$ 0.35 m (%)	$\le$ 0.40 m (%)	Right hand $L_2$ (m)				Intention accuracy (%)
Seconds	1	1.5	2	2.5	2.5	2.5	1	1.5	2	2.5	2.5
RNN [2]	0.404	0.702	0.81	0.784	4.12	15.09	0.308	0.536	0.745	0.598	–
$\Box$ REE	0.206	0.352	0.385	0.399	82	96	0.188	0.325	0.452	0.365	–
$\Box$ Obstacles											
$\Box$ Intention											
REE	0.189	0.343	0.479	0.383	58	66	0.135	0.240	0.335	0.268	–
$\Box$ Obstacles											
$\Box$ Intention											
$\Box$ REE	0.185	0.321	0.336	0.358	72	72	0.187	0.326	0.403	0.363	–
obstacles											
$\Box$ Intention											
$\Box$ REE	0.108	0.184	0.260	0.209	**100**	**100**	0.074	0.127	**0**.**134**	**0**.**142**	**90**.**2**
$\Box$ Obstacles											
Intention											
REE	0.105	0.178	0.261	**0**.**208**	**100**	**100**	0.073	**0**.**122**	0.176	0.143	–
obstacles											
$\Box$ Intention											
REE	0.111	0.183	0.197	0.210	**100**	**100**	**0**.**071**	0.131	0.176	0.141	83.33
$\Box$ Obstacles											
intention											
$\Box$ REE	**0**.**106**	**0**.**172**	**0**.**186**	**0**.**208**	**100**	**100**	0.076	0.129	0.142	0.145	83.33
Obstacles											
Intention											

Again, the results in Table 2 follow the leave-one-out methodology.

#### 3D Human Motion Prediction Experiments

Given the seasonal nature of the harvesting operation, real-world testing of the model in an actual scenario has not been conducted yet. However, the model’s correctness and performance have been extensively evaluated using the training and validation datasets. To ensure a comprehensive evaluation, approximately 15% of the data is left out of the training dataset and used for validation purposes. This approach allows us to assess how well the model generalizes to unseen data.

The evaluation process follows a similar methodology as the handover operation, allowing for a fair comparison between the two tasks. The primary evaluation metric used is the $L_2$ distance between the model’s predictions and the ground truth for the entire upper body. Although the model now predicts the entire human body skeleton, including the legs, we focus on the upper body difference to facilitate comparison with the previous results obtained in the handover experiments.

By using the validation dataset, we can gauge the model’s accuracy and how well it generalizes to new scenarios and variations in human motion. The goal of this evaluation is to ensure that the model’s predictions are accurate and reliable, even in scenarios not encountered during training. While real-world testing is essential to validate the model’s performance in an authentic environment, the rigorous evaluation on the validation dataset provides a solid basis for the model’s correctness and effectiveness in predicting human motion during the harvesting operation.

The results obtained from the harvesting task evaluation, as shown in Table 2, require further explanation. Firstly, it is important to note that the analysis of the phase could only be conducted using the harvesting phase data. Other phases of the harvesting task did not involve direct collaboration with the robot (such as Approach cluster, Move Away, Gesture, and Approach Box), making the accuracy of the predictions in those phases less meaningful. The only exception is the Drop Cluster phase. However, the analysis of this phase was not considered due to the short length of the sequences, which made it challenging for the predictions to reach at least 25 frames for a fair comparison. Additionally, the close proximity of the box to the robot camera caused the pose estimator to fail during the actual drop, further complicating the analysis.

The most significant conclusion drawn from the results is that creating a specific model for the particular phase (Harvest phase) yields the most accurate results across all the mentioned metrics. The body accuracy drops to around 20 cm, and all of the predicted skeletons show an error under 35 cm with respect to the ground truth. The accuracy for the human right hand (the one holding the scissors) is around 14 cm.

**Fig. 11:**
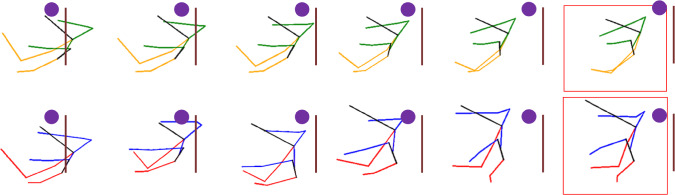
Sequence of the harvest phase during the harvest operation. The prediction properly follows the ground truth skeleton. The position of the grape cluster is marked with a purple circle, while the vineyard trunk is represented just as an aproximation to clarify the plot. In this sequence, the harvester is backwards and he is walking away from the robot

**Fig. 12:**
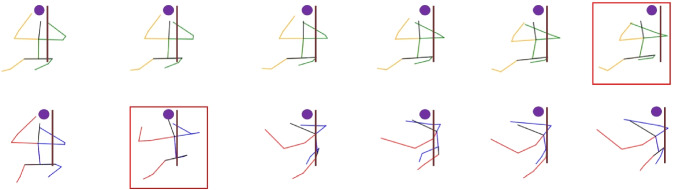
Sequence of the harvest phase during the harvest operation. Top: Sequence predicted. Bottom: Ground truth sequences. The predicted sequence doesn’t turn to the left as much as the ground truth, but the model is able to turn to the same direction than the ground truth. The position of the grape cluster is marked with a purple circle, while the vineyard trunk is represented just as an aproximation to clarify the plot. In this sequence, the harvester is backwards and he is harvesting the cluster

Interestingly, it was observed that for the harvesting phase, context and intention did not significantly improve the results. This can be explained by the fact that during the harvest, the humans do not move very much, except for their arms while performing the cutting. Therefore, adding context in this case did not provide substantial additional value. Context is typically more useful when the human needs to move around the scene with different goals in mind. Similarly, the intention prediction did not show significant improvement because almost all the frames during this phase correspond to a collaborative intention, except for some of the last frames when the human may start moving away, indicating an “adversarial” intention. However, the model trained specifically for the harvesting phase did not have enough data to learn how to classify the sequences, leading it to classify all frames as “collaborative” intention to maximize the metric.

Nevertheless, when the model was trained with all the data, and not just the harvesting phase data, adding context did improve the accuracy, especially for the human hand. The addition of context allowed the model to improve the prediction of the human hand by almost 10 cm, reaching an accuracy of 0.268 cm.

Furthermore, incorporating the intention prediction into the general model slightly improved the accuracy of the whole body, reducing the error from 39.9 to 35.8 cm. Moreover, it provided valuable information about the intention that the human may exhibit in the future, which is a critical output in human–robot collaboration scenarios.

**Fig. 13:**
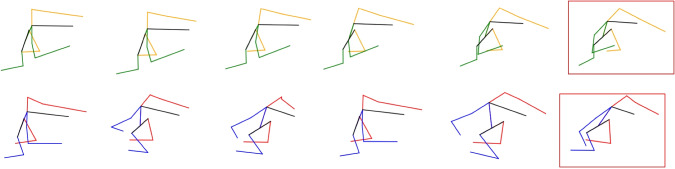
Sequence of the drop phase during the harvest operation. The prediction properly follows the ground truth skeleton. In this sequence, the harvester is sideways looking at the right side. He is dropping the collected grapes into the robot’s box. Top row: predicted skeletons, yellow color represent the left side of the human, while green represents the right side. Bottom row: ground truth skeletons, red color represent the left side of the human, while blue represents the right side. In both cases, black represents the spine and head of the human

**Fig. 14:**
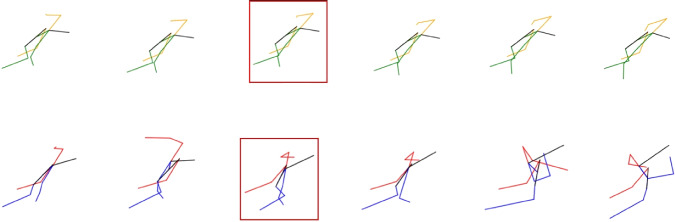
Sequence of the drop phase during the harvest operation. The predicted sequence is able to follow the movement of the human at the beginning of the phase. During the last frames, the ground truth motion starts raising after dropping the bunches, which the models fails to predict. In this sequence, the harvester is sideways looking at the right side. He is dropping the collected grapes into the robot’s box. Top row: predicted skeletons, yellow color represent the left side of the human, while green represents the right side. Bottom row: ground truth skeletons, red color represent the left side of the human, while blue represents the right side. In both cases, black represents the spine and head of the human

Some of the output sequences from the model can be seen in Figs. 11, 12, 13, 14.

## Conclusion

In this study, we have presented a human motion prediction model that incorporates contextual information and human intention to enhance human–robot interactions in handover and harvesting tasks. The results of our experiments show that the combination of context and intention significantly improves the accuracy of the predictions, leading to more successful and natural interactions between humans and robots.

In the handover experiments, we conducted real robot tests to evaluate the impact of the prediction model on human–robot interactions during handover operations. The participants’ feedback revealed that when the robot was capable of predicting human motion and intentions, the overall interaction experience was perceived as better. The participants felt that the robot’s movements were more natural, and they felt more secure and comfortable during the handover process.

Regarding the intention conditioning of the model, while it may not have resulted in a substantial increase in positional accuracy of the samples, the ability to generate multiple predicted options holds valuable potential. This feature might enable the robot to anticipate and adapt to various potential scenarios during its interaction with humans. We intend to delve deeper into this aspect in our future research endeavors.

For the harvesting task, we further explored the impact of context and intention detection on the prediction model’s performance. The results indicated that using context and intention detection for specific phases of the task significantly improved the accuracy of the model. Additionally, by detecting the human’s future intention, we gained the ability to anticipate whether the human would continue with the interaction or not, which is valuable in complex tasks like harvesting.

Overall, our findings suggest that enabling the robot to predict human motion and intentions is highly beneficial for enhancing collaboration between humans and robots in various scenarios. The use of context and intention not only improves the accuracy of the predictions but also leads to more natural and successful human–robot interactions, ultimately enhancing the usability and acceptability of the robot. These insights pave the way for further advancements in human–robot collaboration and contribute to the development of more capable and intuitive robots in real-world applications.

## Data Availability

The datasets generated during and/or analysed during the current study are available from the corresponding author on reasonable request.
